# Role of Prognostic Nutritional Index and C-Reactive Protein/Albumin Ratio in Prognosis of Locally Advanced Nasopharyngeal Carcinoma

**DOI:** 10.3390/medicina62020377

**Published:** 2026-02-14

**Authors:** Taliha Güçlü Kantar, Tolga Doğan, Burçin Çakan Demirel, Melek Özdemir, Semra Taş, Bedriye Açıkgöz Yıldız, Gamze Serin Özel, Ceren Mordağ Çiçek, Burcu Yapar Taşköylü, Atike Gökçen Demiray, Serkan Değirmencioğlu, Arzu Yaren, Gamze Gököz Doğu

**Affiliations:** 1 Department of Medical Oncology, Denizli State Hospital, Denizli 20070, Türkiye; dr_tolgadogan94@yahoo.com (T.D.); melekozdemir@hotmail.com.tr (M.Ö.); 2 Department of Medical Oncology, School of Medicine, Medipol University, Istanbul 34214, Türkiye; brcn_ckn@hotmail.com; 3 Department of Medical Oncology, School of Medicine, Pamukkale University, Denizli 20070, Türkiye; semratasdr@gmail.com (S.T.); bedriyeacikgoz09@gmail.com (B.A.Y.); gamze_239@hotmail.com (G.S.Ö.); cerenmordag@gmail.com (C.M.Ç.); drburcuyapar@gmail.com (B.Y.T.); gokcenakaslan@gmail.com (A.G.D.); arzu_yaren@yahoo.com (A.Y.); ggd2882@gmail.com (G.G.D.); 4 Department of Medical Oncology, Denipol Hospital, Denizli 20010, Türkiye; drserkandeg@hotmail.com

**Keywords:** prognostic nutritional index (PNI), C-reactive protein/albumin ratio (CRP/Alb), nasopharyngeal carcinoma, systemic inflammation response

## Abstract

*Background and Objectives:* Nasopharyngeal carcinoma (NPC) is a distinct type of head and neck cancer with unique epidemiological and pathological characteristics. Inflammatory and nutritional markers have been increasingly recognized as prognostic indicators in cancer. In this study, we aim to evaluate the significance of the prognostic nutritional index (PNI) and C-reactive protein/albumin ratio (CRP/Alb) in the prognosis of patients with locally advanced nasopharyngeal carcinoma (NPC). *Materials and Methods:* We retrospectively analyzed a total of 78 patients diagnosed with locally advanced NPC who received chemoradiotherapy (CCRT) with or without induction or adjuvant chemotherapy between January 2010 and April 2024. Patient characteristics, treatment modalities, inflammatory and nutritional markers, overall survival (OS), and progression-free survival (PFS) were assessed. Kaplan–Meier survival analysis and Cox regression models were used to evaluate the prognostic impact of PNI and CRP/Alb. *Results*: A lower PNI (≤52.90) and lower CRP/Alb ratio (≤0.14) were significantly associated with higher mortality risk (*p* = 0.043 and 0.023, respectively). Tumor size (≥32.85 mm) was also found to be a significant prognostic factor (*p* = 0.041). Patients receiving CCRT alone or with adjuvant chemotherapy had a better OS and PFS compared to those who received induction chemotherapy plus CCRT (*p* = 0.028 and 0.002, respectively). Multivariate Cox regression analysis indicated that CCRT + AC (HR: 0.17, *p* = 0.029) and CCRT (HR: 0.25, *p* = 0.049) significantly reduced the risk of death. *Conclusions*: PNI and CRP/Alb ratio are independent prognostic markers in locally advanced NPC, providing valuable insights into patient stratification and treatment optimization. These findings support their integration into routine clinical practice for risk assessment. Future large-scale, multicenter studies are warranted to confirm these findings.

## 1. Introduction

Nasopharyngeal carcinoma (NPC) is a distinct malignancy that exhibits unique epidemiological patterns, with particularly high incidence in East and Southeast Asia. It differs from other squamous cell carcinomas of the head and neck in terms of epidemiology, histology, natural history, and response to treatment. Globocan 2023 data estimates that nasopharyngeal carcinoma accounts for over 133,000 new cases and 80,000 deaths worldwide annually, highlighting its significant global burden [1]. In endemic populations, risk factors are Epstein–Barr virus (EBV) infection, environmental factors (such as high consumption of preserved foods and smoking), and genetic predisposition, while alcohol and tobacco use, which are also classic risk factors for other head and neck tumors, are also associated with the etiology. Patients may remain asymptomatic for a long time. This is thought to originate from the rosenmuller fossa, an anatomically hidden region of the nasopharynx, and most patients present with locally and/or regionally advanced disease due to this long asymptomatic period or, in some cases, due to a missed diagnosis [2].

The main treatment modality for NPC is radiotherapy (RT), but the use of intensity-modulated radiotherapy (IMRT) has significantly improved the cure rate. Concurrent chemoradiotherapy (CCRT) is the standard treatment approach in all stages except stage 1 and has improved local recurrence-free and overall survival (OS). However, the incidence of distant metastasis is still high [3]. The chemotherapy (CT) regimen to be administered, the addition of induction and/or adjuvant CT to CRT, and the optimal treatment sequence are still under investigation.

Although the tumor lymph node metastasis (TNM) staging system is considered the most valuable prognostic factor for NPC in clinical practice, it is limited in determining prognosis and making the right treatment choices due to the heterogeneity of patients in the same stage who often have different risk factors. Studies have shown an association between inflammatory markers and poor prognosis in patients with various tumor types [4]. The use of hematologic markers as indicators of systemic inflammation, including neutrophil, lymphocyte, and platelet counts, expressed alone or as a ratio, has been found to be associated with cancer prognosis [5]. Recent studies have drawn attention to the prognostic importance of the relationship between immunonutritional status and tumor [6]. The patient’s choice of treatment and quality of life are affected by their nutritional status and immune function. Therefore, monitoring and determining the patient’s pre-treatment nutritional and immune status play important roles in determining the curative effects of treatment and the prognosis of the disease. The prognostic nutritional index (PNI) calculated from serum albumin concentration and peripheral blood lymphocyte count may be a valid indicator of the immune and nutritional status of the patient [7]. Serum inflammatory markers such as C-reactive protein (CRP) or albumin levels have been reported to be prognostic markers in malignancies in addition to inflammatory markers based on blood cell counts [8,9]. Systemic inflammatory markers such as C-reactive protein may be influenced not only by tumor-related inflammation but also by upper respiratory tract infections, chronic sinusitis, odontogenic infections, nutritional status, and cancer-related weight loss. Therefore, careful patient selection and interpretation of inflammatory biomarkers are essential in oncological studies.

Despite advances in treatment, NPC remains a significant global health burden due to late-stage diagnosis and high rates of distant metastasis. In previous studies, prognostic factors in NPC have not been adequately determined and the role of nutrition and inflammation in prognosis has not been fully elucidated.

In this study, we analyzed the prognostic values of PNI and CRP/Alb ratio markers before treatment in locally advanced NPC patients.

## 2. Materials and Methods

### 2.1. Patient Data

Data on seventy-eight patients with local advanced NPC who received chemoradiotherapy ± induction/adjuvant chemotherapy from January 2010 to April 2024 were collected and analyzed. NPC was diagnosed based on histological evidence.

All patients were administered concurrent chemoradiotherapy (CCRT), with the chemotherapy regimen comprising cisplatin (40 mg/m^2^ weekly) and the adjuvant chemotherapy (AC) regimens including PF (cisplatin 80 mg/m^2^ + 5-fluorouracil 3000 mg/m^2^) and TPF (docetaxel 60 mg/m^2^ + cisplatin 60 mg/m^2^ + 5-fluorouracil 3000 mg/m^2^) every 21 days after CCRT, for at least one cycle. The induction chemotherapy regimens included gemcitabine plus cisplatin (GP).

All patients were analyzed in terms of age at diagnosis, gender, body mass index, stage, pre-treatment complete blood count parameters and systemic immune-inflammation index, overall survival, and progression-free survival. The tumors were staged according to the American Joint Committee on Cancer (AJCC, 8th ed., 2017) TNM staging system [10].

Blood samples used for calculating PNI and CRP/Alb ratios were obtained prior to initiating any treatment, after excluding active local/systemic infections. Baseline body weight and body mass index were recorded at the same pre-treatment time point.

Inclusion Criteria

- Patients with NPC who received concurrent chemoradiation combined with or without adjuvant chemotherapy.
- Age ≥ 18 years.
- Patients with an ECOG performance status of 0–1.
- Availability of complete pre-treatment laboratory parameters including full blood count, albumin, and CRP.
- Documented TNM staging (AJCC 8th edition).
- Blood samples obtained within 1–3 days before treatment initiation.
- Complete clinical follow-up information available for survival analysis.

Exclusion Criteria

- Multiple cancers at diagnosis.
- TNM stages I and IVb.
- Active local or systemic infections at the time of blood sampling (acute tonsillitis, sinusitis, dental/periodontal infection, pneumonia).
- Chronic inflammatory or autoimmune diseases and hematologic malignancy.
- Chronic liver disease, cirrhosis, chronic kidney disease, or severe heart failure.
- Incomplete clinical or biochemical data.

### 2.2. Calculated Parameters

The following prognostic scores were calculated using laboratory values:

PNI = [Serum albumin (g/dL) × 10] + [Lymphocyte count (/nL) × 0.005].

CRP/Alb ratio = CRP/Albumin.

### 2.3. Statistical Analysis

Statistical analyses were performed using “IBM SPSS Statistics for Windows. Version 25.0 (Statistical Package for the Social Sciences, IBM Corp., Armonk, NY, USA)”. Descriptive statistics are presented as n and % for categorical variables and mean ± SD and median (min–max) for discrete variables. ROC Curve analysis was used for mortality prediction by various indices, and the Kaplan–Meier method was used to compare survival and PFS times between clinical groups. Finally, multivariate cox regression results of various clinical variables on mortality risk are presented. *p* < 0.05 was considered statistically significant.

## 3. Results

### 3.1. Patient Characteristics

The mean age of the 78 patients included in this study was 51.74 ± 14.13 years with a median of 53 years (range of 19–75 years), and 37.2% of the participants were 50 years of age or younger and 62.8% were over 50. When gender distribution was analyzed, 70.5% were female and 29.5% were male, and the mean body mass index (BMI) was 26.59 ± 5.30 with a median of 25.90 (range 17.40–44.40). While the rate of smoking was 59.0%, 41.0% of the participants were non-smokers, while with alcohol consumption, 94.9% of the individuals stated that they did not drink alcohol and 5.1% stated that they did. Epstein–Barr virus (EBV) positivity was detected in 66.7%, the rate of negativity was 5.1%, and EBV status was unknown in 28.2%. P16 positivity was found in 5.1%, negativity in 15.4%, and unknown status in 79.5% (Table 1). In terms of T stage, T1 was observed in 44.9%, T2 in 25.6%, T3 in 23.1%, and T4 in 6.4%. In terms of N stage, N0 was observed in 6.4%, N1 in 30.8%, N2 in 57.7%, and N3 in 5.1%. Regarding stage, 23.1% of the patients were in stage 2, 65.4% in stage 3, and 11.5% in stage 4. The mean PET-CT primary lesion SUV uptake was 11.94 ± 5.46 with a median of 11.06 (range of 3.56–29.75) (Table 1).

### 3.2. Survival Outcomes

In terms of treatment modalities, 37.2% of patients received induction chemotherapy (IC) + concomitant chemoradiotherapy (CRT), 42.3% received CRT + adjuvant chemotherapy (AC), and 20.5% received only CRT. A total of 19.2% of patients exited and 24.4% progressed (Table 1).

Median overall survival (months) was not available, but the overall survival (months) according to treatment groups was statistically significant (*p* = 0.028) (Table 2). Overall median PFS (months) was not reached, but the median PFS (months) according to treatment groups was statistically significant (*p* = 0.002) (Table 3).

### 3.3. Prognostic Factors

The estimates of CRP/Alb (*p* = 0.023), PNI (*p* = 0.043), and tumor size (*p* = 0.041) parameters were statistically significant in discriminating the presence of mortality (Table 4). The first treatment variable was found to be significant in univariate analyses (Table 2). Age, CRP/Alb, PNI, and tumor size variables were included in the multivariate model considering the *p* < 0.250 rule. According to the results of the model, it was determined that the CCRT + AC arm (HR: 0.17; 95%CI: 0.03–0.83; *p* = 0.029) and the CCRT arm (HR: 0.25; 95%CI: 0.66–0.99; *p* = 0.049) reduced the risk of death (Table 5).

## 4. Discussion

In this study, we collected data on pre-treatment systemic inflammatory and nutritional markers (including BMI, PNI, and CRP/Alb) in patients with NPC to examine their clinical and prognostic value and compare their predictive accuracy. We found that low PNI and low CRP/Alb were prognostic markers in discriminating mortality.

**Figure 1:**
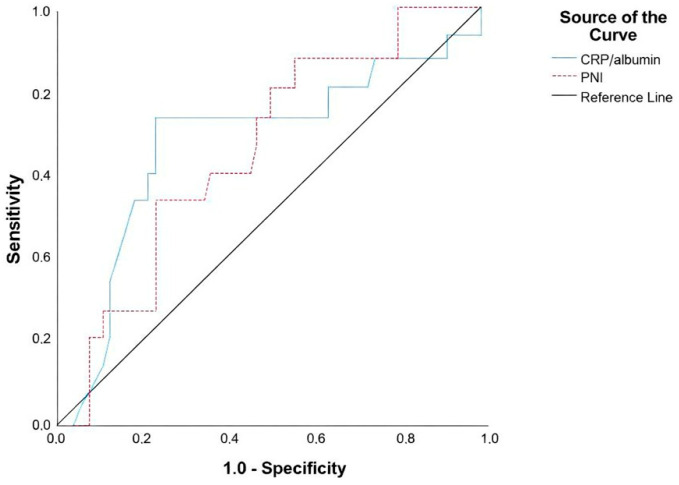
ROC analysis in CRP/Alb and PNI.

The key role of inflammation in tumor cell proliferation, resistance to apoptosis, escape from the immune system, new angiogenesis, distant metastasis, and treatment resistance has been demonstrated in an increasing number of studies [11]. Furthermore, some studies have shown that nutrition and immune status play a decisive role in tumor microcirculation and progression [12]. As a systemic nutritional and immune index, PNI is an objective and convenient index reflecting nutritional immune status. Although studies on the prognostic value of PNI in NPC are limited, studies in head and neck cancers suggest that PNI is a prognostic marker [6,13,14]. In the present study, we defined the median value of PNI (52.90) as the cut-off value, which is consistent with those in previous multiple studies (range: 50–52) [15,16]. In addition, as a result of the evaluation performed with ROC analysis, it was observed that the PNI had diagnostic value in predicting the presence of mortality (AUC: 0.669; 95%CI: 0.523–0.815; *p* = 0.043) (Table 4) (Figure 1).

There have been three meta-analyses investigating the prognostic value of PNI in NPC. Each of these meta-analyses included approximately 10 studies and evaluated a large number of patients. These studies revealed that low PNI is a risk factor for worse OS, PFS, and distant metastasis-free survival (DMFS) [17,18,19]. In NPC specifically, meta-analytic evidence supports the prognostic value of PNI, with low PNI associated with worse overall and metastasis-free survival [17]. More recent single-center data from the IMRT era further corroborate that baseline PNI provides independent prognostic information and can be strengthened when combined with other biomarkers (e.g., LDH) [20]. In addition to survival prediction, recent work suggests that PNI may relate to treatment response endpoints such as lymph node regression following CCRT, reinforcing its potential value for pre-treatment risk stratification [21]. Low PNI may indicate impaired immune function due to lymphopenia, potentially compromising tumor surveillance and facilitating disease progression.

Inflammation-based scores, including the CRP/Alb ratio, capture the systemic inflammatory response and catabolic state and have shown prognostic relevance across multiple solid tumors [8,9]. There is increasing evidence that the CRP/Alb ratio is an independent inflammation marker in various cancer types and may be a more accurate prognostic indicator than many inflammation markers. High CRP/Alb ratio is associated with poor prognosis in many studies. While high CRP levels reflect inflammation and tumor progression, low albumin levels may indicate malnutrition and systemic inflammation [22,23]. In NPC, large retrospective cohorts and a dedicated meta-analysis have generally been associated with a higher CRP/Alb ratio with worse survival and higher risk of distant failure [24,25]. However, in our study, low CRP/Alb ratio was found to be a poor prognostic value in mortality discrimination (AUC: 0.669; 95%CI: 0.523–0.815; *p* = 0.023) (Table 4) (Figure 1). This apparent discordance could be explained by several, non-mutually exclusive factors. First, CRP is highly time-sensitive and may reflect transient intercurrent processes; differences in blood sampling timing relative to symptom burden, subclinical infection, or corticosteroid/nonsteroidal anti-inflammatory drug use can influence baseline values. Second, patients with advanced disease and cancer-related immune dysfunction may present with a blunted acute-phase response despite poor prognosis, potentially producing lower CRP values even in aggressive disease. Third, cut-off selection and cohort composition (endemic vs. non-endemic patterns, stage distribution, and treatment heterogeneity) can invert associations when the marker is near the assay lower limit or when albumin is relatively preserved. These hypotheses merit prospective validation with standardized sampling and adjustment for confounding comorbidities.

The reason for this is that Epstein–Barr virus (EBV)-positive nasopharyngeal cancer is generally associated with good responses to treatment and CRP levels may increase in EBV-positive patients due to inflammatory processes. Similarly, a low CRP/Alb ratio may reflect an anti-inflammatory tumor microenvironment and may have a poor impact on treatment response. Recent studies have shown that pre-treatment plasma EBV DNA has been used clinically in recent years for diagnosis, risk stratification, monitoring, and prediction of NPC prognosis [26,27,28]. In a retrospective and prospective large-scale cohort study, it was found that EBV-DNA or hs-CRP levels alone were positively correlated with DFS and OS [29]. Notably, newer metastatic-disease data suggest that dynamic CRP behavior during treatment—when interpreted alongside EBV DNA clearance—may better reflect treatment response and prognosis than a single baseline value, particularly in the modern chemoimmunotherapy era [17]. Taken together, our findings support the concept that one-time baseline CRP/Alb may be insufficient in capturing the complex interaction between host inflammation, EBV biology, and treatment effect and that longitudinal biomarker trajectories deserve study.

In our study, we found the predictive value of tumor size in ROC analysis for the prognostic factors predicting mortality (*p* = 0.041) (Table 4). We also found a better 2-year and 5-year PFS (*p* = 0.002) and OS (*p* = 0.028) in the CCRT arm compared to the IC + CCRT and CCRT + AC arms (Table 2 and Table 3). In our study, we found that early-stage NPC had a better prognosis and survival in line with the literature. According to Cox regression analysis results, the CCRT + AC arm (*p* = 0.029) and CCRT arm (*p* = 0.049) decreased the risk of death (Table 5). The reason for this is that induction chemotherapy was administered in patients with high tumor burden, large primary tumors T3–T4, N2–N3, and bulky nodal involvement. Treatment selection remains a key determinant of survival in NPC. CCRT is the standard backbone for locoregionally advanced disease, and international guidance endorses multimodality strategies with systemic therapy integrated with radiotherapy, tailored to risk and stage [30,31]. In recent years, treatment intensification strategies have evolved, including optimized induction regimens and adjuvant approaches for high-risk groups. For example, a contemporary phase 3 trial in N2–3 disease demonstrated improved progression-free survival with adjuvant cisplatin–gemcitabine compared with cisplatin–fluorouracil after CCRT, supporting adjuvant escalation in selected high-risk patients [32]. Furthermore, contemporary reviews emphasize a move toward individualized systemic therapy, incorporating biomarkers and patient-level risk to guide induction/adjuvant choices and the growing role of immunotherapy in recurrent/metastatic settings [33].

Clinically, our data suggest that baseline PNI and CRP/Alb ratio—readily available from routine blood tests—may help identify patients who warrant closer surveillance, early nutritional intervention, and proactive supportive care to maintain treatment intensity. However, given the unexpected directionality observed for CRP/Alb, external validation is essential before clinical implementation. Future prospective studies should standardize sampling time points, evaluate longitudinal changes, include detailed comorbidity and medication data, and ideally integrate EBV DNA kinetics and modern treatment regimens to develop and validate multivariable prognostic models.

This study has limitations inherent to its retrospective design, including potential selection bias, missing/unmeasured confounders (e.g., dietary habits, vaccination status, and recent viral infections such as COVID-19), and heterogeneity in staging work-up and treatment. Biomarker cut-offs were derived from our dataset and may not generalize across populations. EBV DNA data were not uniformly available and thus could not be incorporated into the primary models. Despite these limitations, the present findings contribute local evidence supporting nutrition- and inflammation-based risk assessment and highlight areas where biomarker interpretation may differ across real-world cohorts.

## 5. Conclusions

According to this study, markers such as PNI and CRP/Alb ratio, which assess inflammation and nutritional status in nasopharyngeal cancer patients, play important roles in predicting disease prognosis. We show that low pre-treatment PNI and CRP/Alb ratio were found to be associated with survival outcomes in patients with locally advanced nasopharyngeal carcinoma. At the same time, when evaluated in terms of treatment strategies, it was observed that adding adjuvant chemotherapy to CCRT provided a survival advantage. However, some variables such as the effect of EBV positivity on inflammation markers need to be further investigated and larger-scale studies are needed.

In conclusion, these biomarkers demonstrate robust potential for routine prognostic stratification in nasopharyngeal carcinoma. External validation in prospective multicenter cohorts and standardization of optimal cut-off values and measurement timings are key steps in supporting clinical implementation.

## Figures and Tables

**Table 1:** Sociodemographic and clinical characteristics (n = 78).

Variables	N	%
**Age**		
Mean	51.74 ± 14.13	
Median (min–max)	53.50 (19–75)	
≤50	29	37.2
>50	49	62.8
**Gender**		
Female	55	70.5
Male	23	29.5
**BMI**		
Mean ± SD	26.59 ± 5.30	
Median (min–max)	25.90 (17.40–44.40)	
**Smoker**		
None	32	41.0
Yes	46	59.0
**Alcohol**		
None	74	94.9
Yes	4	5.1
**EBV**		
Negative	4	5.1
Positive	52	66.7
Unknown	22	28.2
**P16**		
Negative	12	15.4
Positive	4	5.1
Unknown	62	79.5
**T classification**		
T1	35	44.9
T2	20	25.6
T3	18	23.1
T4	5	6.4
**N classification**		
N0	5	6.4
N1	24	30.8
N2	45	57.7
N3	4	5.1
**Stage**		
2	18	23.1
3	51	65.4
4a	9	11.5
**Pet CT Primary lesion SUV uptake**		
Mean ± SD	11.94 ± 5.46	
Median (min–max)	11.06 (3.56–29.75)	
**Treatment**		
IC + CCRT	29	37.2
CCRT + AC	33	42.3
CCRT	16	20.5
**CRP/Alb**		
>0.14	51	65.4
≤0.14	27	34.6
**PNI**		
>52.90	44	56.4
≤52.90	34	43.6
**Tumor size**		
<32.85	44	56.4
≥32.85	34	43.6
**Mortality**		
Alive	63	80.8
Ex	15	19.2
**Progression**		
None	59	75.6
Yes	19	24.4
**Follow-up period (month)**		
Mean ± SD	60.58 ± 46.20	
Median (min–max)	45.96 (3.57–196.43)	

**Table 2:** OS comparisons of patients.

OS (Month)	2 Years %	5 Years %	Median (95%CI)	*p*
**Overall**	93.0	83.8	- (-)	
**Gender**				
Male	92.0	83.6	- (-)	0.394
Female	95.2	84.2	- (-)	
**Age**				
≤50	96.3	96.3	- (-)	0.158
>50	91.1	77.6	- (-)	
**Treatment**				
IC + CCRT	82.2	70.0	136.3 (0.0–274.1)	**0.028**
CCRT + AC	100.0	89.2	- (-)	
CCRT	100.0	100.0	- (-)	
**CRP/Alb**				
>0.14	91.2	91.2	- (-)	0.067
≤0.14	96.2	73.1	136.3 (0.0–274.1)	
**PNI**				
>52.90	100.0	93.3	136.3 (-)	0.220
≤52.90	83.8	71.4	- (-)	
**Tumor size**				
<32.85	97.6	91.8	- (-)	0.073
≥32.85	86.6	73.0	- (-)	

Kaplan–Meier curve, log rank test; *p* < 0.05 is statistically significant.

**Table 3:** PFS comparisons of patients.

PFS (Month)	2 Years %	5 Years %	Median (95%CI)	*p*
**Overall**	78.6	73.2	- (-)	
**Gender**				
Male	74.0	65.9	- (-)	0.140
Female	90.0	90.0	- (-)	
**Age**				
≤50	77.0	66.3	- (-)	0.613
>50	79.5	76.9	- (-)	
**Treatment**				
IC + CCRT	61.1	52.4	65.2 (10.4–119.9)	**0.002**
CCRT + AC	89.1	89.1	- (-)	
CCRT	92.9	85.1	- (-)	
**CRP/Alb**				
>0.14	76.8	69.6	- (-)	0.743
≤0.14	81.3	77.4	- (-)	
**PNI**				
>52.90	85.2	79.3	- (-)	0.218
≤52.90	69.4	64.8	- (-)	
**Tumor size**				
<32.85	82.6	79.4	- (-)	0.247
≥32.85	73.1	64.7	- (-)	

Kaplan–Meier curve, log rank test; *p* < 0.05 is statistically significant.

**Table 4:** Analysis of the predictive values of various parameter values in differentiating mortality (ROC analysis for the prognostic factors predicting mortality).

Variables	AUC	95%CI	Cut-Off	Sensitivity (%)	Specificity (%)	*p*
**CRP/Alb**	0.690	0.516–0.863	≤0.14	73.3	74.6	**0.023**
**PNI**	0.669	0.523–0.815	≤52.90	60.0	60.3	**0.043**
**Tumor SUV max**	0.602	0.409–0.794	≥11.64	60.0	64.2	0.310
**Tumor size**	0.671	0.533–0.809	≥32.85	60.0	60.3	**0.041**

AUC, area under the curve; 95%CI, confidence interval; *p* < 0.05 is statistically significant.

**Table 5:** Multivariate Cox regression results of mortality risk of various clinical variables.

OS (Month)	HR (95%CI)	*p*
**Age**		
≤50	Ref.	0.178
>50	2.96 (0.61–14.16)	
**Treatment**		**0.029**
IC + CCRT	Ref.	
CCRT + AC	0.17 (0.03–0.83)	**0.029**
CCRT	0.25 (0.66–0.99)	**0.049**
**CRP/Alb**		
>0.14	Ref.	0.084
≤0.14	2.94 (0.86–9.98)	
**PNI**		
>52.90	Ref.	0.178
≤52.90	2.13 (0.70–6.41)	
**Tumor Size**		
<32.85	Ref.	0.232
≥32.85	1.93 (0.65–5.68)	

−2 log likelihood = 92.18, *p* = 0.012; *p* < 0.05 is statistically significant.

## Data Availability

The data in this study are available from the corresponding author upon reasonable request.
